# Bridging the Gap between Advancements in the Evolution of Diagnosis and Treatment towards Better Outcomes in Achalasia

**DOI:** 10.1155/2019/8549187

**Published:** 2019-02-06

**Authors:** Seng-Kee Chuah, Chee-Sang Lim, Chih-Ming Liang, Hung-I Lu, Keng-Liang Wu, Chi-Sin Changchien, Wei-Chen Tai

**Affiliations:** ^1^Gastrointestinal Motility Unit, Division of Gastroenterology, Kaohsiung Chang Gung Memorial Hospital, College of Medicine, Chang Gung University, 833 Kaohsiung, Taiwan; ^2^Department of Hepatology, Hospital Selayang, Lebuh Raya Selayang-Kepong, Batu Caves, Selangor, Malaysia; ^3^Department of Surgery, Kaohsiung Chang Gung Memorial Hospital, 833 Kaohsiung, Taiwan

## Abstract

Over the past few decades, there was an encouraging breakthrough in bridging the gap between advancements in the evolution of diagnosis and treatment towards a better outcome in achalasia. The purpose of this review is to provide updated knowledge on how the current evidence has bridged the gap between advancements in the evolution of diagnosis and treatment of esophageal achalasia. The advent of high-resolution manometry and standardization based on the Chicago classification has increased early recognition of the disease. These 3 clinical subtypes of achalasia can predict the outcomes of patients, and the introduction of POEM has revolutionized the choice of treatment. Previous evidence has shown that laparoscopic Heller myotomy (LHM) and anterior fundoplication were considered the most durable treatments for achalasia. Based on the current evidence, POEM has been evolving as a promising strategy and is effective against all 3 types of achalasia, but the efficacy of POEM is based on short- and medium-term outcome studies from a limited number of centers. Types I and II achalasia respond well to POEM, LHM, and PD, while most studies have shown that type III achalasia responds better to POEM than to LHM and PD. In general, among the 3 subtypes of achalasia, type II achalasia has the most favorable outcomes after medical or surgical therapies. The long-term efficacy of POEM is still unknown. The novel ENDOFLIP measures the changes in intraoperative esophagogastric junction dispensability, which enables a quantitative assessment of luminal patency and sphincter distension; however, this technology is in its infancy with little data to date supporting its intraoperative use. In the future, identifying immunomodulatory drugs and the advent of stem cell therapeutic treatments, including theoretically transplanting neuronal stem cells, may achieve a functional cure. In summary, it is important to identify the clinical subtype of achalasia to initiate target therapy for these patients.

## 1. Introduction

Achalasia happens due to the absence of peristalsis and is a lower esophageal sphincter (LES) disorder that equally affects both sexes and all ethnicities [1, 2]. It is one of the rare primary motility dysfunctions of the esophagus that has no curative treatment. In patients with susceptible genetic backgrounds (HLA DQA1*∗*0103, HLA DQB1*∗*0603 alleles), virus-induced autoimmune-mediated ganglionitis has been proposed to trigger a cascade of events leading to the selective loss of inhibitory neurons of the myenteric plexus, in return inducing an imbalanced production of acetylcholine (Ach)/nitric oxide (NO) and hence unopposed excitation of the lower esophageal sphincter (LES) [3, 4]. Common presentations of achalasia include gradual dysphagia to both liquid and solid foods, chest fullness, and heartburn. In addition, food regurgitation due to dysphagia can cause pulmonary complications such as chronic cough, choking at night, and aspiration pneumonia. Consequently, chronic food regurgitation will lead to gradual weight loss.

The advent of high-resolution manometry diagnoses and predicts the outcome of achalasia. Concurrent utilization of peroral endoscopic myotomy (POEM) has been rapidly evolving and hence bridging the gap between advancements in the evolution of diagnosis and treatment towards a better outcome in achalasia. In this review, we provide updated knowledge to bridge the gap between advancements in the evolution of diagnosis and treatment of esophageal achalasia to optimize treatment outcomes.

## 2. Evolution in Diagnosis

**Figure 1:**
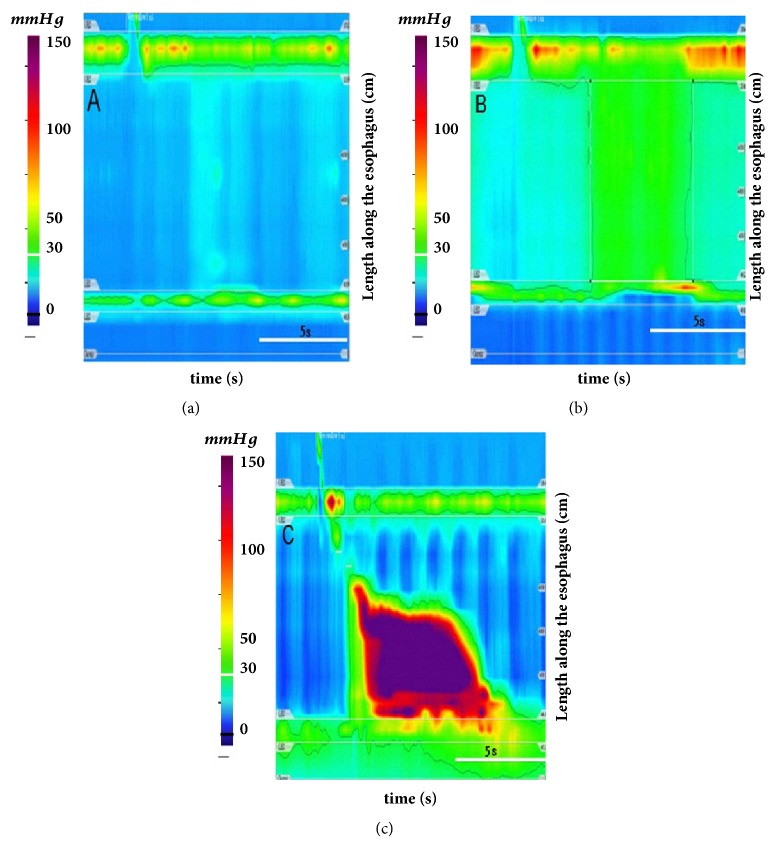
**Chicago classification of achalasia based on HRMPT**. (a) Type I (classic achalasia) refers to patients with absent peristalsis, no pressurization within the esophageal body, and high integrated relaxation pressure (IRP). (b) Type II (achalasia with compression) refers to patients with absent peristalsis and contractile activity, panesophageal pressurization greater than 30 mmHg, and a high IRP. (c) Type III (spastic achalasia) is associated with absent peristalsis and 2 or more spastic contractions with or without periods of compartmentalized pressurization and a high IRP.

Traditionally, achalasia was diagnosed based on commonly used investigations including barium esophagography, esophageal manometry, and endoscopy. An atonic and dilated esophageal body with a classical “bird-beak” appearance of the gastroesophageal junction on a barium swallow and fluoroscopy are typical radiological features. Furthermore, an absence of peristalsis in the esophageal body and absent or abnormal swallowing relaxation of the LES are important criteria for diagnosis with conventional manometry. These traditional studies were not sensitive, with interpretation pitfalls. For instance, it is hard to distinguish artifacts from an actual relaxation-induced swallowing impairment. Moreover, the absence of peristalsis in esophagus is not synonymous with the absence of pressurization within the tubular esophagus. Fortunately, the gap has been bridged since the advent of high-resolution manometry (HRM) and pressure topography [5]. Once combined, these technologies are called high-resolution esophageal pressure topography (HREPT) [6], and they have taken over the role of diagnosing achalasia [5]. Due to the availability of more pressure sensors (22–36) at much shorter intervals (1–2 cm), HRM facilitates a more convenient and comprehensive evaluation of esophageal motor function than conventional manometry. Among the crucial parameters obtained by HRM, the four-second integrated relaxation pressure (IRP-4s), defined as the average lowest pressure through the EGJ for four contiguous or noncontiguous seconds within the relaxation window, can reliably measure LES relaxation and identify esophageal disorders related to EGJ outflow obstruction, especially achalasia. Standardization of diagnosis based on the Chicago classification (Figure 1) has increased the early recognition of this disease [6, 7]. Ever since the Chicago classification was initiated, the diagnosis and management of achalasia has been refined. Moreover, the utilization of esophageal pressure topography has improved the diagnostic accuracy and enhanced the early recognition of clinically relevant subtypes of achalasia, allowing treatment plans to be tailored according to the different subtypes to improve the outcome. In a recent multicenter randomized trial enrolling 247 patients with unexplained dysphagia, HRM demonstrated better diagnostic accuracy for achalasia than conventional manometry. The diagnostic accuracy for achalasia was noted to be more sensitive in the HRM arm than in the conventional manometry arm (26% vs. 12%, P <0.01). Moreover, based on the diagnostic algorithm of the Chicago classification for esophageal motility disorders, achalasia can be further categorized with HRM into three clinical subtypes (Figure 1): type I, absent peristalsis with minimal esophageal pressurization; type II, absent peristalsis with esophageal compression (panesophageal pressurization); and type III, absent peristalsis with distal esophageal spastic contractions. These subtypes are closely related to treatment outcomes after medical or surgical therapies, and type II achalasia has the most favorable outcome [6]. Therefore, the application of HRM in clinical practice can not only improve the diagnostic accuracy but also enhance the early recognition of clinically relevant subtypes of achalasia to tailor the treatment plan and improve the outcomes of achalasia patients. However, we should always attempt to rule out pseudoachalsia. In these situations, computed tomography (CT) or endoscopic ultrasound (EUS) could be useful tools in addition to conventional endoscopy [4].

## 3. Treatment Evolution: From Conventional Treatment to Peroral Endoscopic Myotomy (POEM) and Endoluminal Functional Lumen Imaging Probe System (ENDOFLIP)

### 3.1. Conventional Treatment

For esophageal achalasia, pharmacological treatment has typically played a minor role because, up to now, the efficacy of the best available medications has remained unsatisfactory. Nitrates can increase cyclic guanosine monophosphate levels and lead to increases in NO concentrations in smooth muscle cells, while calcium channel blockers (CCBs) are useful for blocking the entry of calcium and cause esophageal muscle relaxation. Under such circumstances, these drugs can reduce the LES pressure by smooth muscle relaxation and eventually relieve dysphagia [21]. Sildenafil exerts a similar effect to CCBs and nitrates [22, 23]; however, intolerable side effects, such as pedal edema, dizziness, and headache, are common; thus, pharmacological treatments are seldom used over the long term.

There are several options for the endoscopic treatment of achalasia. First, endoscopic local injection of botulinum toxin into the LES muscle of patients with achalasia lowers LES tone and could lead to symptom resolution. Paralysis of both voluntary and involuntary muscles can be achieved by administrating botulinum toxin [24], which is a potent biological toxin that acts at the presynaptic cholinergic nerve terminal by inhibiting acetylcholine release from nerve endings, leading to restoration of the balance between inhibitory and excitatory neurotransmitters and thus normalizing the unopposed excitation of the LES. This treatment option for achalasia is safe and has few complications [24]. Excellent immediate responses have been reported in some studies, with success rates of more than 90% [24]. Significant improvement in esophageal functions, such as increased esophageal diameter, decreased LES pressure, and improvement in the transit time by scintigraphy, is observed after botulinum toxin injection (BTI). Generally, complications due to BTI therapy for achalasia are mild, as the dosage is too small to induce serious adverse effects such as generalized paralysis. The common side effects reported include transient chest pain and reflux symptoms. The main shortcoming of BTI is its shorter duration than other treatments, as it normally lasts for 6−9 months. The highest success rates are found in elderly patients and in patients with an LES pressure not exceeding the upper limit of normal prior to treatment [25]. Elderly and high-risk patients with concomitant comorbid disease might not be suitable for other standard treatment modalities. Under such circumstances, BTI is a viable option.

Conventional endoscopic balloon dilations cause mechanical tears in the muscle fibers of the LES. The Rigiflex dilator is the commonly used dilator with a fully inflated diameter that usually ranges from 3 cm or more to achieve maximal pressure for a satisfactory result. Dilations can be performed by either a fluoroscopy-guided procedure [12, 13, 26] or endoscopy-guided procedure [9, 14]. Dilation sessions and the inflation time needed for a successful dilation vary between patients and are operator dependent. A larger dilator may be used in a single dilation session for patients who have relapsed based on symptom scores [11]. In most available case series, immediate and short-term results have reportedly been favorable [8, 10, 11, 14] (Table 1). However, unfavorable recurrences in fluoroscopy-guided PD patients were reported in large-scale long-term follow-up investigations [12, 13, 27]. Thus, some authors have proposed that long-term remission could possibly be achieved by applying the “on-demand” strategy with repeated PD based on symptom recurrence [4]. PD was found to be relatively safe with uncommon major complications. The main complication was esophageal perforations, and the incidence rate of perforation was approximately 0−2% [27, 28]. Reflux symptoms after PD can usually be controlled with proton pump inhibitors.

Table 2 shows the cumulative effectiveness of surgical myotomy for achalasia. Surgery offers satisfactory long-term results (75-97%) with myotomy of the LES and a concomitant antireflux procedure in minimally invasive LHM with a variety of fundoplication procedures to reduce postoperative reflux [15, 29–31]. Most surgeons generally choose a length of myotomy of 4−5 cm into the esophagus and 2−3 cm into the stomach during LHM [17]. Currently, for most patients with achalasia, surgeons preferably perform minimally invasive LHM with a variety of fundoplication procedures, especially partial fundoplication, as 360° fundoplication can cause more dysphagia [16, 17, 29]. Some randomized controlled trials have shown that the addition of an antireflux procedure to a myotomy substantially reduces the postsurgical incidence and severity of pathological reflux [15, 18, 19]. Overall, postsurgical complications are rare (< 4%) [19, 20]. The reported incidence of a major complication in LHM was approximately 5−10% in esophageal perforation. Some surgeons choose robotically assisted Heller myotomy (RAHM), but the cost is high [32, 33]. Ultimately, if all treatment modalities are unsuccessful, esophagectomy can be considered in patients with recurrent, disabling symptoms or severe complications such as malignancy due to achalasia.

### 3.2. POEM and ENDOFLIP

POEM was introduced as a natural orifice transluminal endoscopic surgery (NOTES). The main advantage of this procedure is that it is incisionless and has good surgical efficacy without surgical morbidity. This novel endoscopic treatment for achalasia was first reported by Pasricha et al. in porcine models [34] and was then popularized by Inoue et al. for patients with achalasia [35]. POEM can be accomplished by creation of a submucosal tunnel at the half of the esophageal circumference, with myotomy beginning 3 cm distal to entry and approximately 7 cm above the gastroesophageal junction. Endoscopically, the inner circular muscle layer of the esophagus is slowly dissected and divided via a small proximal opening of the esophageal mucosa. In this way, POEM can accomplish a longer myotomy than surgical myotomy. By contrast, in patients with advanced disease and severe fibrosis, surgeons can have difficulties extending the length of the myotomy to the thoracic esophagus when performing LHM. Once the myotomy is completed, clipping is important to prevent complications. Numerous reports on this technique have been published, and all of them showed good short-term results without serious complications; however, long-term follow-up results, such as morbidity and postprocedural GERD, are still lacking [35–47]. A meta-analysis of 27 studies involving 2065 patients showed a clinical success rate of 98% at 3 months in maintaining an Eckardt score < 3 [46]. This meta-analysis has proven that POEM has a good short-term outcome; however, these studies had some limitations, as they were short-term studies of less than 12 months' duration, and long-term data were lacking. Hence, validation of the long-term safety and durability of this procedure could make POEM a breakthrough in the treatment of esophageal achalasia, and this can be achieved by a long-term prospective study. To evaluate immediate clinical results, a recent single-center study was conducted involving 318 patients. Stavropoulos et al. reported a clinical success of 95% at 2 years, and it was maintained for up to 3 years [47]. Inoue et al. reported the largest series, a cohort of 500 POEM patients, and found a significant reduction in Eckardt scores and LES pressures at 2 months, 1 year, and 3 years after the procedure (88%) [48].

Although POEM is a promising new treatment, most endoscopists worldwide have not yet mastered this technique, as POEM is a very technically demanding procedure with a steep learning curve. Even for an experienced endoscopist, POEM can be challenging. A few authors have even suggested that it is best reserved for patients who are expected to have technically difficult LHM, such as those with prior major abdominal surgery, prior LHM, and morbid obesity [49]. Zhou et al. reported that POEM achieved short-term symptom relief in > 90% for cases of failed Heller myotomy [45]. In a multicenter study of 1872 patients, overall, there were 156 adverse events reported in 7.5% of patients [50]. Multivariable analysis results demonstrated that the usage of a triangular tip knife (OR=3.22, P < 0.05) and the use of an electrosurgical current other than spray coagulation (OR=3.09, P< 0.05) were significantly associated with the occurrence of adverse events. Other important parameters that were associated with adverse events were a sigmoid-type esophagus (OR=2.28, P < 0.05), followed by an endoscopist experience level of less than 20 cases (OR= 1.98, P< 0.05). Overall, this large study concluded that POEM was a relatively safe procedure with zero mortality if performed by an expert in an experienced high-volume center. Nevertheless, the evolution of treatments with the utilization of POEM has changed the landscape for the treatment of achalasia. There is now an increased array of management options in addition to traditional therapy.

ENDOFLIP (Crospon Medical Devices, Galway, Ireland) measures the changes in intraoperative esophagogastric junction (EGJ) dispensability, which enables a quantitative assessment of luminal patency and sphincter distension; however, this technology is in its infancy with little data to support its intraoperative use. It is a system consisting of a balloon electrode (BE) and a functional imaging luminal probe (FLIP) that uses impedance planimetry for real-time measurement of the diameter of the EGJ. BE measures the EGJ distensibility (cross-sectional area/luminal pressure) during volume-controlled distension, while FLIP provides a useful measurement of the EGJ distensibility in achalasia patients that is correlated with symptom severity [51]. When used intraoperatively, it can provide dynamic real-time information. With the advent of ENDOFLIP, it is possible to measure the EGJ diameter before and after the procedure, and it can be particularly useful to detect cases of incomplete myotomy before finishing the surgery to drive therapy. Emerging studies have shown that intraoperative distensibility measurements were correlated with postoperative outcomes; thus, it is a meaningful calibration tool for ensuring that an adequate myotomy is performed intraoperatively [52, 53]. Some authors have proposed that intraoperative FLIP measurements are predictors of treatment outcomes. The measurement of EGJ distensibility is complementary to existing tests, suggesting a potentially important role in the clinical management of achalasia. Teitelbaum et al. used ENDOFLIP to measure the effect of variable distal myotomy lengths on EGJ distensibility intraoperatively during the POEM procedure; they concluded that myotomy extension across the LES complex and to 2 cm into the gastric wall led to normalization of EGJ distensibility, whereas subsequent extension to 3 cm distal to the EGJ did not increase compliance further [53]. ENDOFLIP could potentially impact therapeutic decision making; however, the proven utility of ENDOFLIP needs further clinical evidence. This technology is currently in its infancy, with little data to support its intraoperative use.

### 3.3. Comparison between Treatment Modalities

#### 3.3.1. LHM versus PD

The main advantage of PD is that it can be performed as an outpatient procedure; on the other hand, the disadvantage of PD is that patients usually require more than 1 treatment session. PD is generally safe, with minimal injury and bleeding; thus, postprocedural gastroesophageal reflux is minimized. Numerous reports have demonstrated that an “on-demand” strategy based on symptom recurrence with repeated PD can optimize clinical success rates to levels that are comparable to those of LHM [54, 55]. Weber et al. demonstrated that both PD and LHM are effective treatment options, but LHM might be more durable [56]. In a European achalasia trial, Rohof et al. compared PD with myotomy in 176 patients who were followed up for 5 years and showed that for type I achalasia, both are efficacious (PD: 81% vs. LHM: 85%), while PD was better than LHM for type II achalasia (PD: 100% vs. LHM: 93%). However, for type III achalasia, PD showed discouraging results (PD: 40% vs. LHM: 86%) [57].

Decisions should be made based on a balance among treatment efficacy, complications, cost effectiveness, and durability of the procedure. Clinical judgment should also consider the risk of perforation due to repeated dilations. The major complication of PD is perforation of the esophagus, which occurs in approximately 0−2% of patients [8, 28]. Conversely, mucosal tears occur in 12% of patients during LHM but can be repaired and recovered. The main drawback of LHM is the incidence of postsurgical acid reflux, which occurs 3-34% of the time [15–18, 29–31]. In comparison, the symptoms of reflux in post-PD patients can be easily controlled by proton pump inhibitors. Long-term complications of reflux, such as stricture, Barrett' s esophagus, and adenocarcinoma, are rare. The experience of the operator is a determinant in treatment success. LHM is recommended for males and for younger patients (< 40 years) as well as patients who fail to respond to 1 or 2 initial dilations [58]. In a cost-effectiveness analysis for achalasia, LHM had a higher initial cost, and PD was a most cost-effective treatment option for adults with achalasia [59]. On the other hand, in the long run, LHM can be cost effective if the durability is greater than 10 years with a single treatment [60]. In general, LHM is a more durable treatment for achalasia, while PD is the first nonsurgical choice and is more cost effective. Concurrent with advancements in the evolution of diagnostic techniques, such as HRM, and treatments, such as POEM and ENDOFLIP, there were high expectations that these new modalities would generate better treatment outcomes that dwarfed the debate over the two conventional methods of LHM and PD.

#### 3.3.2. POEM versus LHM

POEM is rapidly evolving, and it is unavoidable that there are ongoing debates on the gold standard treatment for achalasia comparing POEM to LHM. A recent meta-analysis that included 4 studies with a total of 317 patients showed that POEM achieved treatment outcomes that were equivalent to LHM in terms of the length of myotomy required, operative time, postprocedural Eckardt score, and complications [61]. In assessing a cost analysis, POEM incurred significantly lower total charges than LHM (USD 14481 vs. USD 17782, P value = 0.017) [62].

Moreover, emerging evidence has shown that patients with type III achalasia have significantly greater relief of symptoms with POEM than with LHM. This effect has been clearly demonstrated in an international multicenter comparative study of 75 patients with type III achalasia. Kumbhari et al. reported that POEM led to improved clinical responses in type III achalasia patients (98% vs. 80.1%), with significantly less adverse events in the POEM group than in the LHM group (6% vs. 27%, P value < 0.01). A recent meta-analysis of uncontrolled POEM series reported a weighted pooled response rate of 92% (95% confidence interval, 84-96%) in type III achalasia with a myotomy length of 17.2 cm [63]. The length of myotomy can be gauged by HRM, esophageal wall thickening on endoscopic ultrasound, or with an intraoperative functional luminal imaging probe. Despite the dissection field adjacent to several vital mediastinal structures, the safety profile of POEM is excellent [50, 64]. Over 7000 cases have been performed to date, and remarkably, no mortalities have been reported. Currently, although there is ongoing research comparing POEM and LHM, information on the long-term efficacy of POEM (5 to 10 years) is still lacking.

GERD is always the main concern for myotomy procedures when treating achalasia. LHM can be performed with Dor fundoplication to minimize the complication of reflux but not with POEM, which results in much higher occurrences of GERD after POEM. Both Salvador's and Alessandro's reports showed that postoperative reflux was 10% or less with LHM, which was lower than the incidence with POEM [65, 66]. It has been reported that endoscopic or pH-metry evidence of post-POEM GERD was found in 58% of patients, including endoscopic esophagitis in 23% (proton pump inhibitor use was uncontrolled) in a multicenter case-control series study of 282 patients [67]. Schlottmann et al. compared the outcomes of POEM (1958 patients) and LHM (5834 patients) and found that POEM was more effective than LHM at relieving dysphagia in the short term (mean followup of 16 months), but it was associated with a very high incidence of pathologic reflux (OR= 9.31 for erosive esophagitis) [68].

The question of whether POEM diminishes in efficacy with time is a concern. Thus, more studies of long-term clinical responses to POEM are needed. Until now, the efficacy of POEM has been based on short- and medium-term outcome studies from a limited number of centers until long-term prospective studies and RCTs comparing POEM to LHM are conducted. The short- and medium-term outcomes of POEM are similar to those of LHM, with the exception that postprocedural GERD occurred more frequently after POEM [62, 67, 68].

#### 3.3.3. POEM versus PD

Wang et al. reported that the short-term and intermediate efficacy of POEM and PD for treating achalasia in patients aged ≥ 65 years were comparable [69]. However, a large-scale, randomized study with long-term followup is necessary in order to make a definitive conclusion. Ponds et al. reported the first randomized trial on POEM vs. PD that enrolled 133 patients with treatment-naïve achalasia (POEM, n=67 and PD, n=66) [70]. At the one-year followup, clinical responses were significantly higher in the POEM group (92.2% vs. 70%, P<0.01). One perforation occurred in the PD group and was treated with endoscopic suturing, while there were no severe adverse events in the POEM group. Endoscopy performed off PPIs revealed significantly a higher incidence of esophagitis in the POEM group than in the PD group (40% grade A/B, 8.3% grade C/D vs. 13.1% grade A/B, 0% grade C/D, p<0.05). The outcomes of patients who underwent POEM in this study are on par with previous published reports. However, the results of PD in this study were worse than those from prior randomized trials, which may be because a disproportionate number of patients with type III achalasia were included in the PD arm. As expected, patients with type III achalasia fared better with POEM than with PD.

## 4. Which Is the Best Option for Treatment?

**Figure 2:**
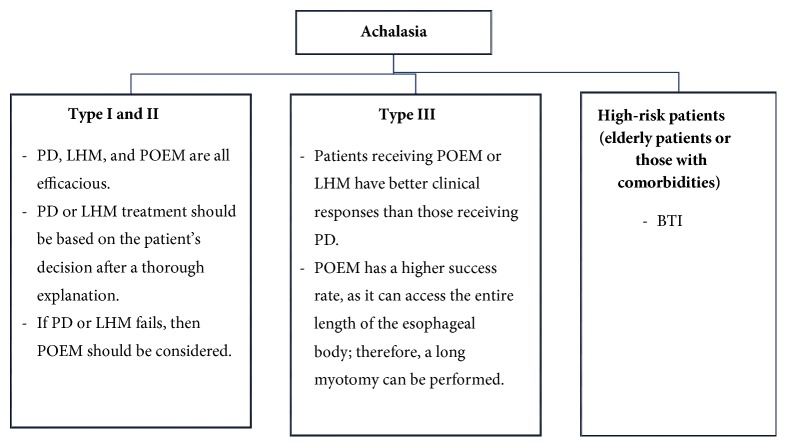
**Proposed treatment algorithm based on the subtype of achalasia**. Decisions should be based on the expertise in the center. PD: pneumatic dilation; POEM: peroral endoscopic myotomy; LHM: laparoscopic Heller myotomy; BTI: botulinum toxin injection.

A proposed algorithm for the treatment of achalasia is shown in Figure 2. Since the introduction of HRM, achalasia could be subdivided into 3 clinical subtypes, which has enabled the clinician to initiate goal-directed treatment according to the achalasia subtype. Types I and II achalasia respond well to POEM, LHM, and PD, while most studies have shown that type III achalasia responds better to POEM or LHM than to PD [71]. However, type III achalasia has a spastic contraction in the mid- and distal esophagus that reduces the pressure of the LES as well as the pressure of the affected spastic segment, which is also important. Therefore, a higher success rate is observed with POEM than with LHM, as the entire length of the esophageal body can be accessed, and therefore, a long myotomy can be performed. Moreover, POEM can be calibrated according to the image seen on HRM.

Because POEM is still evolving, and not all centers are equipped with an experienced operator, LHM and PD remain the crucial treatment option in these centers. BTI is known to increase the risk and the difficulties of subsequent LHM [72]. Under such circumstances, the judicious selection of patients for BTI is vital. It is a suitable alternative for the minority of patients who have concomitant comorbidities and are thus deemed to be at a high risk [24, 25]. Although BTI is not a preferred treatment, in certain patients with tortuous megaesophagus and previous failed pneumatic dilatations, BTI is surprisingly efficacious. On the other hand, BTI is less effective than PD and LHM for sustained symptomatic relief in patients with achalasia. While the short-term efficacy of BTI therapy is comparable to that of PD, Ghoshal et al. reported a high rate of relapse during the first year of followup [73].

### 4.1. Future Perspectives

At present, three clinical trials are ongoing to assess the efficacy of treatment modalities for achalasia. These trials are summarized in Table 3. These trials will be completed by the end of 2018 through 2023. These exciting clinical studies could determine the future direction of treatment modalities for achalasia once completed. Clinical research is now heading towards the possibility of an infectious event associated with certain genetic factors that trigger autoimmune mechanisms, thus affecting neurons, as a possible etiology of achalasia. It has been postulated that the identification of an immunomodulatory drug in the future could be a possible treatment target for achalasia. With the advent of stem cell therapeutic treatment, theoretically transplanting neuronal stem cells for a functional cure might be a great achievement in the future. Future work should consider the potential of stem cell therapy.

## 5. Summary

Over the past few decades, there have been encouraging breakthroughs in bridging the gap between advancements in the evolution of diagnosis and treatment towards a better outcome in achalasia. The advent of HRM in the diagnosis of achalasia has been a great advantage, as it is sensitive and easy to perform. The standardization of diagnosis based on the Chicago classification has increased early recognition of the disease. These 3 clinical subtypes of achalasia can predict the outcomes of patients, and the introduction of POEM has revolutionized the choice of treatment. Based on current evidence, POEM is a promising strategy that is effective against all 3 types of achalasia, but the efficacy of POEM is based on short- and medium-term outcome studies from a limited number of centers. Types I and II achalasia respond well to POEM, LHM, and PD, while most studies have shown that type III achalasia responds better to POEM than to LHM and PD. In general, among the 3 subtypes of achalasia, Type II achalasia has the most favorable outcomes after medical or surgical therapies. Thus, clinicians must initiate goal-directed therapy and individualized treatment by scrutinizing treatment efficacy, complications, and expertise among centers. Quantitative assessment of luminal patency and sphincter distension has been realized by innovations such as ENDOFLIP. This innovative technique could potentially facilitate future assessments and treatments for achalasia, but it is currently in its infancy, with little data to support its intraoperative use.

## Figures and Tables

**Table 1:** Cumulative effectiveness of pneumatic dilators for the treatment of achalasia.

Author (year)	Article type	No. of patients	Type of dilator (size, cm)	Success (%)	Mean follow-up (yrs.)
Vela (2006) [8]	Retrospective study	106	3.0-4.0	28-62	3.2

Chuah (2009) [9]	Prospective study	32	3.0	69-91	4.5

Hulsemans (2010) [10]	Retrospective study	209	3.0-4.0	72	5.8

Tanaka (2010) [11]	Retrospective study	55	3.0-3.5	74.5	2.3

Gupta (2017) [12]	Retrospective study	72	3.0-4.0	60-91%	3

Müller (2018) [13]	Retrospective study	107	3.0-4.0	36-64%	13.8

Lee (2018) [14]	Prospective study	29	3.0-3.5	96.6%	1.5

**Table 2:** Cumulative effectiveness of surgical myotomy for achalasia.

Author (year)	Article type	No. of patients	Type of surgery	Success (%)	Mean follow-up (yrs.)
Balakrishna (2015) [15]	RCT	62	LMH-Dor or LMH-AOH (Angle of His accentuation)	98.38	1.8

Campos (2009) [16]	Meta-analysis	3086	LMH, LHM-Nissen	89	2.9

Falkenback (2003) [17]	RCT	10	LHM-Nissen	70	8.0

Rebecch (2008) [18]	RCT	138	LHM-Nissen/Dor	85-97	10.4

Rawlings (2012) [19]	RCT	60	LHM-Dor/Toupet	90.9-93.1	1.0

Parise (2011) [20]	Retrospective study	137	LHM-Dor	90	5.4

LHM: laparoscopic Heller myotomy; RCT: randomized control trial.

**Table 3:** Ongoing clinical trials.

**Topic**	**ID**	**Study design**	**Estimated date of completion **
POEM vs. BTI in spastic esophageal disorders	*NCT02663206*	RCT	Jul 2020

Multicenter study comparing endoscopic pneumodilation and POEM	*NCT01793922*	RCT	Jan 2023

Endoscopic versus laparoscopic myotomy for the treatment of idiopathic achalasia: a randomized, controlled trial (POEM rcpmt)	*NCT0160678*	RCT	Dec 2018

POEM: peroral endoscopic myotomy; RCT: randomized controlled trial; BTI: botulinum toxin injection; LHM: laparoscopic Heller myotomy.
